# Analysis of the Effect of Conditions of Preparation of Nitrogen-Doped Activated Carbons Derived from Lotus Leaves by Activation with Sodium Amide on the Formation of Their Porous Structure

**DOI:** 10.3390/ma14061540

**Published:** 2021-03-21

**Authors:** Mirosław Kwiatkowski, Xin Hu

**Affiliations:** 1 Department of Fuel Technology, Faculty of Energy and Fuels, AGH University of Science and Technology, al. A. Mickiewicza 30, 30-059 Krakow, Poland; 2 College of Chemistry and Life Sciences, Zhejiang Normal University, Jinhua 321004, China; huxin@zjnu.cn

**Keywords:** adsorption, micropores, carbonization, chemical activation, activated carbons

## Abstract

This paper presents results of the analysis of the impact of activation temperature and mass ratio of activator to carbonized precursor *R* on the porous structure of nitrogen-doped activated carbons derived from lotus leaves by carbonization and chemical activation with sodium amide NaNH_2_. The analyses were carried out via the new numerical clustering-based adsorption analysis (LBET) method applied to nitrogen adsorption isotherms at −195.8 °C. On the basis of the results obtained it was shown that the amount of activator, as compared to activation temperatures, has a significantly greater influence on the formation of the porous structure of activated carbons. As shown in the study, the optimum values of the porous structure parameters are obtained for a mass ratio of *R* = 2. At a mass ratio of *R* = 3, a significant decrease in the values of the porous structure parameters was observed, indicating uncontrolled wall firing between adjacent micropores. The conducted analyses confirmed the validity of the new numerical clustering-based adsorption analysis (LBET) method, as it turned out that nitrogen-doped activated carbons prepared from lotus leaves are characterized by a high share of micropores and a significant degree of surface heterogeneity in most of the samples studied, which may, to some extent, undermine the reliability of the results obtained using classical methods of structure analysis that assume only a homogeneous pore structure.

## 1. Introduction

The rapid growth of industrialization and urbanization is resulting in an increase in the level of pollution emitted to the natural environment. Therefore, many measures are being taken and new technologies are being developed to stop this disastrous trend for humanity. One of the materials successfully used for many years for harmful substances removal has been activated carbons [1,2]. The common use of activated carbons is associated with their large specific surface area and adsorption capacity, good mechanical properties, ease of regeneration and relatively low production costs [3]. In addition, the surface of activated carbons is hydrophobic, which allows for adsorption processes from aqueous solutions and gaseous streams with a high degree of humidity.

The large increase in interest in activated carbons in recent centuries and the increase of research to expand their manufacture processes are due both to increased needs in traditional fields of application and the development of new technologies based on the use of these materials. Adsorption processes from the liquid phase on activated carbons are used, among others, in the treatment of water intended for food, in the food industry and in the treatment of wastewater from industrial processes [4]. In turn, adsorption processes from the gaseous phase on activated carbons are used in the treatment of gaseous streams, in the separation of gaseous mixtures, in the recovery of solvents and in natural gas and hydrogen storage processes [5].

Activated carbons are manufactured in the process of physical activation preceded by carbonization or in the process of chemical activation. However, in most cases, the raw material requires pre-treatment before it can be activated by crushing and screening it into the appropriate grain fraction, and, in some cases, a water or acid cleaning stage is used to remove impurities from the raw material and the impurities mixed with it.

Physical activation involves two successive stages. The first is the carbonization of the raw material, carried out at a high temperature usually 500–1000 °C, in an inert gas atmosphere, usually nitrogen or argon, or in a slightly oxidizing atmosphere [6]. During this process, the least durable chemical bonds present in the structure of the raw material break, and as a result of progressive polymerization and polycondensation, a homogeneous mass of the products of carbonization with a high content of elemental carbon is formed. In addition, the gases and vapors emitted during carbonization contribute to the formation of the original pore system in the resulting products of carbonization. The second stage is the activation of the obtained products of carbonization by activation at a high temperature in the range of 800–1000 °C in the presence of an oxidizing agent such as water vapor, carbon dioxide or mixtures of these activators [7].

Chemical activation, in turn, is a one-stage process in which the raw material, after being impregnated or mixed with an activating agent, is subjected to high temperatures in an inert gas atmosphere [8]. The chemical compounds used in the chemical activation process are most often phosphoric acid (V) [9,10], potassium hydroxide [11,12], zinc chloride [13] and recently, sodium amide [14,15,16].

The activation process time depends on the final temperature, the activator used and the expected pore size. Activation usually leads to weight loss based on a carbonized product from 10% to 40%. When the weight loss is less than about 10%, the pore development is insufficient and insufficient performance cannot be prepared due to the insufficient pore volume. When weight loss is greater than about 40%, the proportion of pores with pore diameters between 0.6 and 0.8 nm decreases due to structure degradation.

The greater reactivity of the activating agents used in the chemical activation process allows not only to skip the carbonization stage, but also to lower the process temperature, and thus reduce energy costs and the time necessary to create a porous structure. In the process of chemical activation, the final product is prepared with high efficiency, characterized by a strongly developed specific surface and porosity, and a very low ash content [17]. The main disadvantage of chemical activation is its high cost, resulting both from the need to use expensive activators as well as the introduction of a stage for removing the excess of activators and by-products by washing. A large-scale chemical activation process in industrial practice would generate additional costs related to waste disposal.

Both activation methods, i.e., both physical and chemical, are used in activated carbon production processes to obtain high-quality adsorbents. For adsorption processes from the gaseous phase, e.g., solvent recovery or removal of traces of volatile organic substances, adsorbents produced via chemical activation have better properties, whereas for liquid phase processes, e.g., water treatment, carbons produced via physical steam activation have better properties. Basic methods for the production of activated carbon can be interconnected in different ways, e.g., chemically activated carbons can be subjected to additional physical activation to modify the pore structure of the final product [18,19,20].

The activated carbons produced via chemical activation are usually more microporous with a predominant proportion of pores less than 2 nm in diameter. In contrast, activated carbons produced by physical activation are usually more mesoporous with a predominant proportion of pores between 2 and 50 nm in diameter [21,22].

The properties of activated carbons depend both on the production methods used and the conditions under which they are carried out as well as the raw material. The choice of a precursor for the production of activated carbons depends on its availability, acquisition costs, original structural structure and purity, and the use of biomass waste from the wood, forestry and food industries is becoming more and more popular [23,24,25].

The arguments for the use of biomass waste for the production of activated carbons include relatively low cost of acquisition, easy accessibility of the raw material and its renewability. From the economic and ecological points of view, the adsorbents obtained from agricultural, forestry and wood industry waste make an alternative to those produced from coals and others precursors. It should also be mentioned that the production of activated carbons from waste biomass is not only one of the alternative methods of utilizing waste, but also a way of converting waste into a valuable and sustainable product [26,27,28,29,30]. Wood processing industry and the carpentry industry and other related industries have a huge potential of raw materials, hence the production of activated carbons from biomass waste could increase economic return and reduce pollution.

It should be stressed that due to the complex structure of biomass and complex mechanisms occurring during carbonization and activation processes affecting the properties of the final product, i.e., activated carbons, in order to obtain its expected porous structure and physicochemical properties, needs comprehensive experimental studies and analyses. In recent years, due to the demand for porous materials with a large specific surface area and high mechanical strength used in environmental protection and power engineering, a dynamic development of research on the processes of production of activated carbons from waste materials of biomass origin has been observed [14,15,16,17,18,19,20,21,22,23,24,25,26,27,28,29,30].

## 2. Materials and Methods

The new adsorption technologies and their need to compete with other technologies require increasingly efficient adsorbents, and simple and cheap physical adsorption methods do not make it possible to obtain them. Therefore, new methods of obtaining them are being sought, and there is great scope for developing chemical activation methods, including the search for new chemical compounds that could be used in the production of highly effective activated carbons, as well as seeking new cheap and readily available raw materials for the production of activated carbons.

A paper by Liu et al. [31] presents the results of a study which yielded nitrogen-doped activated carbons prepared from lotus leaves by carbonization and chemical activation with sodium amide NaNH_2_. The activated carbons were obtained according to different ratios of the mass of the activator to the char, i.e., *R* = 1, 2, and 3. The activation temperature was set to 450, 500, or 550 °C. A comprehensive description of the experiment, including the methods of obtaining nitrogen-doped activated carbons, was presented in the previous publication [31]. These nitrogen-doped activated carbon samples were labeled as LLAC/*T*; *R*, with *T* indicating the activation temperature and *R* the ratio mass of the activator, i.e., sodium amide NaNH_2_ to carbonized lotus leaves. In the analysis of adsorption isotherms in the mentioned study, popular methods, i.e., Brunauer—Emmett–Teller (BET) [32], the T-plot [33], the Dubinin–Raduskevich (DR) [34] and the Density Functional Theory (DFT) [35,36,37,38] were used.

In the study of activated carbon production processes, a particularly important issue is to assess the influence of various factors on the formation of a porous structure, for which precise characteristics of the porous structure are necessary. The main component of activated carbons is elemental carbon, whose proportion is usually in the range of 85% to 95% by weight. The rest consists of elements such as hydrogen, nitrogen, sulfur and oxygen as well as a mineral substance.

The physicochemical properties of activated carbons depend on their texture, which can be considered as a system of randomly arranged graphite-like crystallites, connected with each other by cross-links, separated by fragments of an amorphous carbonaceous substance with a low degree of ordering and by a mineral substance derived from the raw material. The crystallites forming the skeleton of activated carbons resemble graphite crystals in terms of structure, but in their case, larger and unequal interlayer spacing, a smaller degree of ordering and the presence of vacancies and gaps in the crystal lattice are observed—which is why the structure of activated carbons is called turbostratic.

The description of the porous structure of carbonaceous adsorbents and adsorption phenomena occurring on their surface is complicated due to the fact that there are different shapes and sizes of pores in the same material and different connections between individual pores. Physical adsorption of various gases has become the most popular method of characterization of microporous and mesoporous activated carbon structures.

Many methods of analyzing the structure of carbonaceous adsorbents based on isotherms of vapor and gas adsorption have been developed, and the most popular are the Brunauer–Emmett–Teller (BET) [32], the T-plot [33], the Dubinin–Raduskevich (DR) [34], and the Density Functional Theory (DFT) [35,36,37,38] methods. However, these methods may often not be sufficient for a precise and reliable analysis of the porous structure of materials with such a complex structure as activated carbons. 

Considering the above, a new series of studies was commenced to an analysis of the impact of activation temperature *T* and mass ratio of activator to carbonized precursor *R* on the porous structure of nitrogen-doped activated carbons prepared from lotus leaves by carbonization and chemical activation with sodium amide NaNH_2_. The analyses were carried out on nitrogen adsorption isotherms via the new numerical clustering-based adsorption analysis (LBET) method.

The LBET method as well as its theoretical basis have been described in the author’s previous publications [39,40,41,42,43,44], therefore, it will not be described in this article. It should be mentioned, however, that the LBET models have five parameters: the volume of the first adsorbed layer *V_hA_* (cm^3^/g), the dimensionless energy parameter for the first adsorbed layer—*Q_A_*/*RT*, the geometrical parameter of the porous structure determining the height of the adsorbate molecule clusters *α*, the geometrical parameter of the porous structure determining the width of the adsorbate molecule clusters *β* and the dimensionless energy parameter for the higher adsorbed layers *B_C_* which can be adjusted by fitting LBET equation to the adsorption isotherm, with a chosen variant of the surface energy distribution function [39,40,41,42,43,44].

## 3. Results

With the use of the new numerical clustering-based adsorption analysis (LBET) method, based on nitrogen adsorption isotherms at −195.8 °C, the following parameters were determined, i.e., *V_hA_* (cm^3^/g), —*Q_A_*/*RT*, *α*, *β*, *B_C_*, as well as additionally: the effective contact correction factor *Z_A_*; the surface heterogeneity parameter *h*; the fitting error dispersion *σ*_e_ and the identification reliability indices *w_id_*. Moreover, also obtained were the adsorption energy distributions on the first layer, for all the activated carbons. These results were compared with computational results obtained from previous studies using the BET, T-plot, DR and DFT methods [31].

## 4. Discussion of the Obtained Results

The results of the earlier calculations carried out via the BET, T-plot and DR methods based on nitrogen adsorption isotherms determined at −195.8 °C [31], collected and presented in Table 1, demonstrate that in the case of activated carbons prepared at *R* = 1, as the activation process temperature values increased, the value of structure parameters increased too, i.e., the *S_BET_* specific surface area calculated by the BET method, the *S_micro_, _T-Plot_* micropore surface area calculated by the T-plot method, and the micropore volume *V_micro DR_*, *V_micro T-Plot_*, *V_micro DFT_* calculated successively using the DR, T-plot and DFT methods as well as the total pore volume *V_total DFT_* calculated by the DFT method. Moreover, a decrease in the value of the *S_ext T-Plot_* parameter was observed, i.e., the size of the external specific surface area calculated by the T-plot method as the temperature of the activation process increases, up to the value of 0 in the case of activated carbon LLAC/550 °C; *R* = 1. On the other hand, for the activated carbon samples prepared with the mass ratio equal to 2, a significant increase in the values of the parameters *S_BET_*, *S_micro T-Plot_*, *S_ext. T-Plot_*, *V_micro T-Plot_*, *V_micro DR_*, *V_micro DFT_* and *V_total DFT_* was observed, in relation to the samples prepared with the ratio *R* = 1 and the increase in the value of these parameters in the case of increasing the activation temperature from 450 °C to 500 °C. However, an increase in the activation temperature, i.e., to 550 °C, resulted in a decrease in the values of the *S_BET_, S_micro, T-Plot_* parameters while increasing the value of *S_Ext. T-Plot_*, *V_micro T-Plot_*, *V_micro DR_*, *V_micro DFT_* and *V_total DFT_* parameters. 

In the case of activated carbons prepared at the mass ratio *R* = 3, lower values of all the parameters determined were observed for the samples prepared at all temperatures in comparison with the samples prepared at the mass ratio *R* = 2, which involves excessive burning of the walls between micropores.

In the case of activated carbon samples prepared at *R* = 3 with temperature increase from 450 °C to 500 °C, an increase in *S_BET_*, *S_micro, T-Plot_*, *S_ext. T-Plot_* parameters and micropore volumes *V_micro T-Plot_*, *V_micro DR_*, *V_micro DFT_* as well as total pore volume *V_total DFT_* was observed. However, further increase of the activation process temperature to 550 °C resulted in the destruction of the porous structure, which manifested itself in a significant decrease of all values of the structure parameters. 

Summing up, the highest value of the *S_BET_* surface area, i.e., *S_BET_* = 1923 m^2^/g, and the specific surface area of the micropores *S_micro T-Plot_*, i.e., *S_micro T-Plot_* = 1772 m^2^/g, was obtained for the activated carbon marked as LLAC/500 °C; *R* = 2, i.e., prepared in the process of chemical activation of NaNH_2_ at temperature of 500 °C. In turn, the largest values of S_ext. T-Plot_, *V_micro T-Plot_*, *V_micro DR_*, *V_micro DFT_* and *V_total DFT_* parameters were obtained for activated carbon LLAC/550 °C; *R* = 2, i.e., prepared at an activation temperature of 550 °C, with a mass ratio of *R* = 2 (*S_ext. T-Plot_* = 210 m^2^/g, *V_micro T-Plot_* = 0.897 cm^3^/g, *V_micro DFT_* = 0.8612 cm^3^/g, *V_total DFT_* = 1.1977 cm^3^/g).

**Figure 1:**
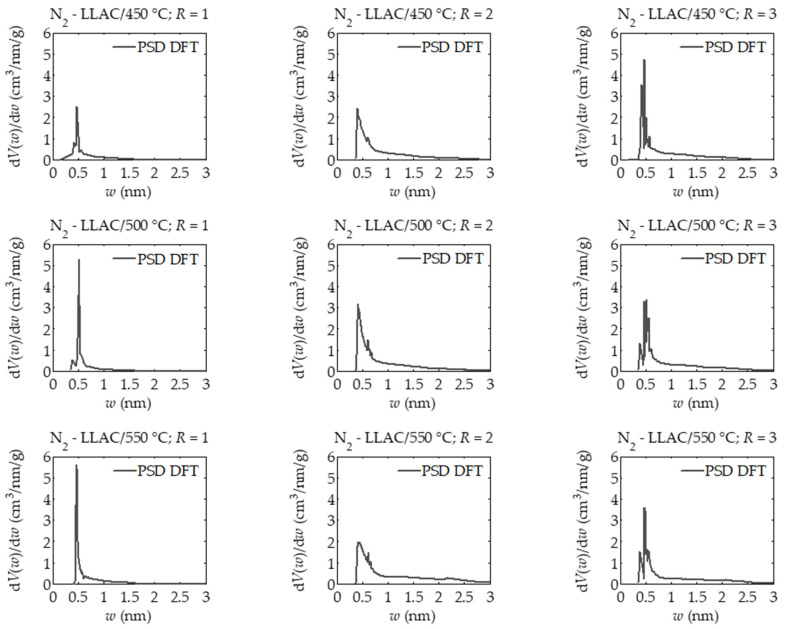
The pore size distributions in the range of 0 to 3 nm determined for all analyzed samples of nitrogen-doped activated carbons prepared from lotus leaves by carbonization and chemical activation with sodium amide NaNH_2_ on the basis of nitrogen adsorption isotherms via the DFT method.

Based on pore size distributions obtained via the DFT method presented in Figure 1, it can be concluded that the samples of activated carbons derived at the activator mass ratio, i.e., sodium amide NaNH_2_ to the lotus leaf carbonization product equal to 1 had a narrow pore size distribution, i.e., from about 0.25 nm to 1.5 nm [31]. The proportion of sub-micropores in the range 0.4 to 0.5 nm for the LLAC/450 °C sample; *R* = 3, was highest.

The shapes of pore size distributions determined for samples prepared at the mass ratio *R* = 2 indicate a significantly higher proportion of both large sub-micropores and micropores and small mesopores, compared to samples prepared at *R* = 1. In the case of activated carbons prepared at *R* = 3, the pore size distribution patterns indicate a higher proportion of micropores. In particular, the pore size distribution patterns determined for LLAC/500 °C; *R* = 2 and LLAC/550 °C; *R* = 3, indicate the predominance of micropores in the range 0.3 to 0.5 nm and 0.7 to 0.75 nm. Therefore, DFT analysis showed a much narrower pore size distribution in the micropore range 0.3 to 0.75 nm determined for the LLAC/550 °C sample; *R* = 3, compared to the LLAC/500 °C sample; *R* = 3.

**Figure 2:**
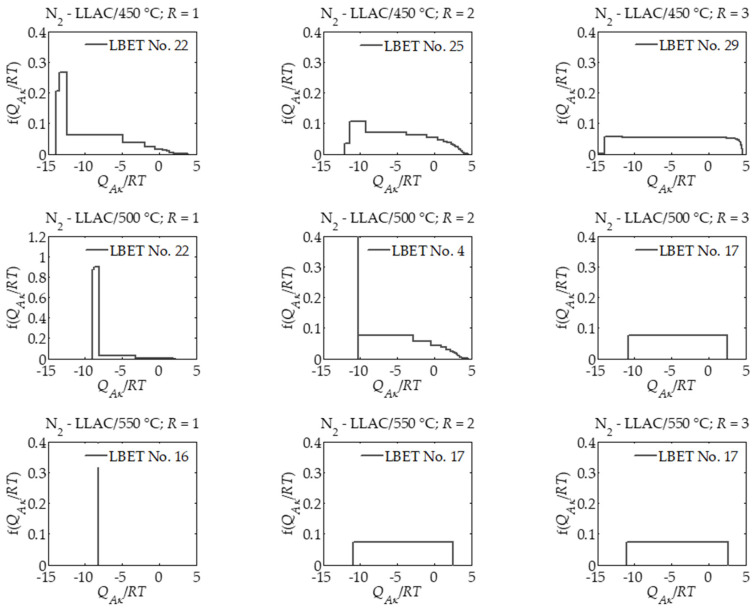
The adsorption energy distributions for the first adsorption layer determined for all analyzed samples of nitrogen-doped activated carbons prepared from lotus leaves by carbonization and chemical activation with sodium amide NaNH_2_ on the basis of nitrogen adsorption isotherms via the LBET method.

However, the results of analyses carried out using BET, T-plot, DR and DFT [31] methods do not fully explain the processes occurring during the preparation of activated carbons under various preparation conditions. Therefore, in this work, the LBET method [39,40,41,42,43,44] was used to analyze nitrogen adsorption isotherms on activated carbons prepared from the products of carbonization of lotus leaves. The results of nitrogen isothermal adsorption isothermal analysis using the above mentioned method are presented in Table 2 and Figure 2. The interpretations were carried out taking into account both the effect of activation process temperature and activator mass ratio to carbonization product *R*.

On the basis of the results of the analysis of nitrogen adsorption isotherms on activated carbon LLAC/450 °C; *R* = 1, i.e., prepared at 450 °C with an activator mass ratio of 1 to the product of the carbonization process, it was shown that this material has an average value of the volume of the first absorbed layer, i.e., *V_hA_* = 0.293 cm^3^/g. 

The values of parameters *Q_A/RT_* and *B_C_* i.e., *Q_A/RT_* = −13.85 and *B_C_* = 4.16, indicate the occurrence of energy constraints on the development of clusters of adsorbate molecules, and the value of the heterogeneity parameter *h* = 5 indicates that the surface area of the material tested is heterogeneous. In the pores of activated carbon LLAC/450 °C; *R* = 1, high and not branched clusters of nitrogen molecules are formed as indicated by the values of geometric parameters, i.e., *α* = 0.70 and *β* = 1.00. Moreover, the number of the matched model indicates that the growth limitations of adsorbate particle clusters are connected with geometric limitations of pores as well as high adsorption energy in layers *n* > 1.

The shape of the distribution of adsorption energy on the surface of the material under study, in turn, indicates the occurrence of a narrow range of sites with high adsorption energy and a wide spectrum of sites with different adsorption energy. Increasing the activation process temperature to 500 °C with the same mass ratio in the case of a sample of activated carbon determined as LLAC/500 °C; *R* = 1, practically did not change the value of the volume of the first absorbed layer, however, the values of energy parameters, i.e., *Q_A_*/*RT* = −9.02 and *B_C_* = 1.14 as well as the parameter of effective contact of adsorbate molecules with the surface of *Z_A_* adsorbent have decreased. The values of geometric parameters *α* and *β* also indicate the formation of low branching clusters of nitrogen molecules in the micropores of the studied material.

A further increase in temperature up to 550 °C, i.e., in the case of the LLAC/550 °C; *R* = 1 sample, resulted in an increase in the volume of the first absorbed layer compared to samples prepared at lower temperatures at the same mass ratio and a decrease in the values of energy parameters. The surface of activated carbon LLAC/550 °C; *R* = 1, was homogeneous as indicated by the value of parameter *h* = 0, and at its pores, high and slightly non-branching clusters of adsorbate particles were formed (*α* = 0.92, *β* = 1.09).

Shapes of adsorption energy distributions on the surface of activated carbons prepared at the mass ratio *R* = 1 indicate that with an increase in activation process temperature, the spectrum of adsorption energy on the surface of the tested materials was significantly narrowed down to the case of LLAC/550 °C; *R* = 1, characterized by an energy homogeneous surface.

Increasing the number of activators to the mass ratio *R* = 2 resulted in a very significant increase in the volume parameter value of the first absorbed layer *V_hA_* and a decrease in the energy parameter value for adsorbed layers higher than the first *B_C_*, the degree of heterogeneity *h* and the geometric parameter *α*. The values of these parameters increased with the activation temperature. A sample of activated carbon LLAC/550 °C; *R* = 2, i.e., the sample prepared at 550 °C at the ratio *R* = 2 had the highest value of *V_hA_* among all analyzed samples, i.e., 1.060 cm^3^/g. Shapes of the distribution of adsorption energy on the surface of activated carbons prepared at a mass ratio *R* = 2 indicate that with the increase in the temperature of the activation process, the unevenly heterogeneous surface of activated carbons with predominant share of high adsorption energy sites became uniformly heterogeneous for the sample prepared at 550 °C.

An increase in the mass ratio *R* to 3 resulted in an increase in the value of the *V_hA_* parameter determined for the LLAC/450 °C; *R* = 3 and LLAC/500 °C; *R* = 3 samples, and for the LLAC/550 °C; *R* = 3 sample prepared at 550 °C, a significant decrease in the value of this parameter was observed. As the activation temperature increased to 500 °C, the values of the energy parameter for the higher adsorbed layers *B_C_* and the degree of heterogeneity of the surface *h*, have significantly decreased. It is worth noting that further increasing the activation temperature to 500 °C did not have a major impact on the *B_C_* and *h* parameter values. The shapes of adsorption energy distributions on the surface of activated carbons prepared at a mass ratio *R* = 3 indicate that the surface of these materials produced at different temperatures was uniformly heterogeneous.

On the basis of the results obtained, it was shown that the amount of activator, as compared to activation temperatures, has a significantly greater influence on the formation of the porous structure of activated carbons. Namely, in the case of nitrogen-doped activated carbons prepared from the products of lotus leaves carbonization in the process of NaNH_2_ activation at 450 °C, a significant increase in the value of the parameter of the volume of the first adsorbed layer, an increase in the value of energy parameters, an increase in the effective contact surface of adsorbate molecules with the adsorbent surface, a significant increase in surface heterogeneity, as well as an increase in the height of clusters of adsorbate molecules were observed together with an increase in the mass ratio *R*.

The shape of the distribution of adsorption energy on the surface of activated carbons prepared from the products of carbonization of lotus leaves in the process of NaNH_2_ activation at 450 °C indicates that with an increase in the value of mass ratio *R*, the energy heterogeneity of the surface of the analyzed samples of activated carbons changes, namely with an increase in the value of mass ratio *R*, the share of places with high adsorption energy decreases.

In the case of the samples prepared at activation temperature of 500 °C, higher values of the volume of the first absorbed layer were observed for the samples prepared at mass ratio *R* = 2 and 3 in comparison with activated carbons obtained at 450 °C, i.e., 0.875 and 0.946 cm^3^/g, respectively, and significantly lower values of energy parameters for the adsorbed layers higher than the first *B_C_* in the case of activated carbons prepared at *R* = 1 and 3. Contrary to the activated carbons prepared at the temperature of 450 °C, in the case of activated carbons prepared at the activation temperature of 500 °C, the degree of surface heterogeneity decreased along with the increase in the mass ratio *R*. Shapes of adsorption energy distributions on the surface of samples produced at the process and activation temperature of 500 °C indicate that with an increase in the amount of activator in relation to the biochar obtained from lotus leaves, the share of high-energy sites was mixed in favor of equally heterogeneous energy distribution of adsorption sites.

In the case of activated carbons prepared at 550 °C, a significant increase in the volume of the first adsorbed layer *V_hA_* as well as in the adsorption energy for the first adsorbed layer *Q_A/RT_* was observed together with an increase in *R* value to 2. The value of the first adsorbed layer volume obtained for LLAC/550 °C; *R* = 2, was the largest of all activated carbon samples analyzed in this study. With a further increase in the amount of activator to *R* = 3 in the case of LLAC/550 °C activated carbon; *R* = 3, however, there was a significant decrease in the value of *V_hA_* parameter to 0.705 with no significant change in other parameters, which indicates micropore wall burning.

Shapes of adsorption energy distributions on the surface of the samples produced at the activation temperature of 550 °C indicate that with an increase in the amount of activator above the mass ratio *R* = 1, the homogeneous pore structure was destroyed to the pore structure characterized by an equally heterogeneous energy distribution of the adsorption sites.

One of the important issues in the analysis of calculated results is the analysis of reliability of the results obtained. The LBET method has built-in tools to assess the reliability and credibility of the results obtained in the form of *σ_e_* matching error dispersion rates and *w_id_* identification coefficient. The values of these indicators determined for all analyzed activated carbons indicate high reliability of identification of adsorption systems as well as high reliability of the designated parameters of porous structure. Such a situation was predictable on the basis of the analysis performed in earlier work [31]. The analyzed micropore structure were practically unimodal, which is indicated by pore size distribution shapes determined by the DFT method with one dominant pore size. Moreover, the values of specific surfaces and micropore volumes indicate that the analyzed material was microporous, i.e., with a dominant share of micropores, which fully meets the theoretical assumptions of LBET class models. However, in the case of analyses carried out using both BET and DR methods [31], the results obtained should be approached with caution because the obtained activated carbons had a high proportion of micropores, which violates the assumptions of the BET theory, as well as the significant degree of surface heterogeneity, which is not taken into account by both BET and DR methods.

## 5. Conclusions

The conducted research has provided also a lot of unique information on the influence of the preparation conditions of activated carbons obtained from lotus leaves and shed new light on the issues related to the assessment of the influence of the preparation conditions of carbon adsorbents on the parameters of porous structure development. On the basis of the results obtained, it was shown that the amount of activator, as compared to activation temperatures, has a significantly greater influence on the formation of the porous structure of activated carbons. As shown in the study, the optimum values of the porous structure parameters are obtained for a mass ratio of *R* = 2. At a mass ratio of *R* = 3, a significant decrease in the values of the porous structure parameters was observed, indicating uncontrolled wall firing between adjacent micropores.

The analysis results obtained with the LBET method showed that the calculation results obtained with the BET method, as well as the DR method, should be approached with great reserve, as the activated carbons obtained had a high proportion of micropores, which violates the basic assumptions of the BET theory. Furthermore, the analyses carried out showed a significant degree of surface heterogeneity in most of the activated carbon samples tested, which could have a negative impact on the reliability of the results obtained, as the BET and DR models, as well as the DFT model, assume only a homogeneous pore structure.

In conclusion, on the basis of the presented results, it can be stated that lotus leaves are not an outstanding material for the production of carbon adsorbents, however, their widespread availability, e.g., in China, as well as negligible acquisition costs make them an interesting and promising raw material for the production of good quality activated carbons. However, to properly exploit the potential of this raw material, it is necessary to precisely select the conditions for the production of activated carbons, and thus to use more advanced tools for the analysis of the porous structure, considering the heterogeneity of the surface and allowing, among others, the drawing of the shape and size of the pores, the adsorption energy on the surface of the adsorbent, as well as the distribution of the energy, and such possibilities are offered by the LBET method.

## Figures and Tables

**Table 1:** The results of the analysis of a microporous structure of nitrogen-doped activated carbons, based on nitrogen adsorption isotherms, determined using the BET [31], T-plot and DR methods.

T (°C)	450			500			550		
*R*	1	2	3	1	2	3	1	2	3
*S_BET_* (m^2^)	792	1650	1557	833	1923	1666	1086	1883	1310
*S_micro, T-Plot_* (m^2^)	710	1520	1481	766	1772	1526	1086	1673	1099
*S_ext. T-Plot_* (m^2^)	82.14	130	75	67	151	140	0.00	210	201
*V_micro T-Plot_* (cm^3^/g)	0.287	0.671	0.637	0.312	0.794	0.719	0.445	0.897	0.556
*V_micro DR_* (cm^3^/g)	0.327	0.710	0.658	0.345	0.788	0.715	0.463	0.818	0.558
*V_micro DFT_* (cm^3^/g)	0.319	0.707	0.655	0.333	0.828	0.722	0.444	0.861	0.580
*V_total DFT_* (cm^3^/g)	0.397	0.819	0.778	0.406	0.974	0.887	0.493	1.197	0.812

**Table 2:** The results of the analysis of a microporous structure of nitrogen-doped activated carbons, based on nitrogen adsorption isotherms, determined using the LBET method.

T (°C)	450			500			550		
*R*	1	2	3	1	2	3	1	2	3
LBET No.	22	25	29	22	4	17	16	17	17
*V_hA_* (cm^3^/g)	0.293	0.764	0.822	0.298	0.875	0.946	0.350	1.060	0.705
*−Q_A_*/*RT*	13.85	12.07	17.10	9.02	10.27	10.81	8.26	10.93	11.12
*B_C_*	4.16	7.67	7.84	1.14	7.03	1.01	1.00	1.00	1.00
*Z_A_*	0.582	0.534	0.670	0.451	0.485	0.500	0.431	0.503	0.508
*h*	5	7	9	5	3	1	0	1	1
*α*	0.70	0.90	1.00	0.16	0.83	0.09	0.92	0.33	0.31
*β*	1.00	1.00	1.00	1.41	1.00	1.00	1.09	1.00	1.00
*σ* *_e_*	0.51	0.59	0.84	0.64	0.47	0.61	0.67	0.73	0.55
*w_id_*	0.06	0.09	0.01	0.07	0.07	0.09	0.02	0.09	0.08

## Data Availability

The data presented in this work can be made available on request.
